# Community understanding of the concept of pre-referral treatment and how this impacts on referral related decision-making following the provision of rectal artesunate: a qualitative study in western Uganda

**DOI:** 10.1186/s12913-018-3209-4

**Published:** 2018-06-19

**Authors:** C. E. Strachan, A. Nuwa, D. Muhangi, A. P. Okui, M. E. H. Helinski, J. K. Tibenderana

**Affiliations:** 1 Malaria Consortium, Development House, 56-64 Leonard Street, London, EC2A 4LT UK; 2 London School of Hygiene and Tropical Medicine, Keppel St, London, WC1E 7HT UK; 3 Cambridge Economic Policy Associates, Queen’s House, 55-56 Lincoln’s Inn Fields, London, WC2A 3LJ UK; 4 Department of Social Work and Social Administration, Makerere University, Kampala, Uganda; 5 National Malaria Control Programme, Ministry of Health, Kampala, Uganda; 6 European and Developing Countries Clinical Trials Partnership, P.O. Box 93015, 2509 AA The Hague, The Netherlands

**Keywords:** Community health worker, Community understanding, Pre-referral treatment, Qualitative, Rectal artesunate, Referral, Severe malaria, Uganda

## Abstract

**Background:**

Successful pre-referral treatment with rectal artesunate (RA) for suspected severe malaria requires operational linkages between community health workers (CHWs) and referral facilities, acceptance of pre-referral treatment and adherence to referral practices by CHWs and caregivers. This qualitative study investigated how community understanding of the concept of ‘pre-referral treatment’ is used in referral related decision-making following provision of RA in Uganda.

**Methods:**

Narrative interviews were conducted with 30 caregivers of children under five who received RA within the previous three months and the 30 associated CHWs who provided the treatment. Nineteen focus group discussions incorporating vignettes from the interviews were held with further caregivers, and 12 with CHWs and women representatives. Twenty traditional healers were targeted for semi-structured interview. Thematic analysis followed a ‘meaning-based’ approach.

**Results:**

CHWs were aware of essential information to be given to caregivers on prescribing RA as indicated by the job aid, specifically urgency for referral, yet there was insufficient emphasis on RA not being a full treatment for severe malaria. Information shared by the CHW appeared to be influenced by the perceived urgency with which the CHW needed to act and the time of day or night the child was seen. Seven of the 32 caregivers did not complete referral post RA administration. Caregivers seemed more likely to adhere to referral advice if they perceived their child’s condition to be severe. Previous caregiver experience and CHW comparisons with Artemisinin-based Combination Therapy (ACT) as a treatment for uncomplicated malaria appeared to raise misperceptions that RA is a complete treatment for severe malaria, thus reducing likelihood to complete referral. CHW implication, or caregiver interpretation, of the need to monitor the child, and some prescription of ACT post RA administration, also confused the need for referral. Both CHWs and caregivers requested further information about the role of RA.

**Conclusions:**

CHW advice should emphasise RA as providing temporary relief prior to facility-based treatment, the importance of referral whether or not a change is seen in the child’s condition, and the dangers of not completing referral. Social behaviour change communication, training and support supervision activities could help promote these messages.

**Electronic supplementary material:**

The online version of this article (10.1186/s12913-018-3209-4) contains supplementary material, which is available to authorized users.

## Background

In cases of suspected severe malaria among children residing in rural areas located far from health facilities, the provision of rectal artesunate (RA) prior to referral can reduce the risk of severe malaria-related mortality and permanent disability [1, 2]. The pre-referral artesunate suppository interrupts disease progression by rapidly reducing the parasite load, usually causing a temporary alleviation of fever and other clinical signs of malaria. However, RA given as pre-referral treatment is not a cure for severe malaria and must be followed by a full dose of anti-malarial treatment and comprehensive management at a health facility [1, 3]. Following World Health Organization (WHO) recommendation of pre-referral RA targeting suspected severe malaria cases among children under 6 years old where intramuscular injections are not available [3], RA has been integrated into national malaria case management policies across high burden malaria countries over the last 5 years.

Successful provision of pre-referral treatment with RA at the community level requires effective linkage and operational referral systems between community health workers (CHWs) and the referral facility. It also requires the acceptance of pre-referral treatment and adherence to referral practices by both CHWs and caregivers of children under five (many countries have used the age cut-off of under 5 years for community service delivery). Existing evidence suggests that the provision of RA at the community level is both feasible as a community intervention and accepted by caregivers, provided that appropriate explanatory information is provided by CHWs [4–7]. Prior knowledge or experience with rectally administered medicines (either traditional or biomedical) has in particular shown to favourably influence acceptability of RA given people are already accustomed to receiving treatment in this way [8, 9]. Studies have indicated that adherence to referral advice after administration of RA is affected by some common factors, including the perceived severity of symptoms and assumed infection, the decision of or permission from the household head to travel for further care, costs associated with referral, alternative demands on caregiver time (i.e. childcare, work in the fields), perceived quality of hospital care, and caregiver recall of referral advice from CHWs [10–12].

Caregiver understanding of the rationale for referral (‘when and why’) has also been found to be key in influencing adherence [10, 13]. This understanding can be further complicated by a change in the child’s condition following RA administration; a study in Tanzania found that caregivers were more likely to adhere to referral advice when the child’s condition failed to improve and less likely when a noticeable improvement was seen [13]. Confusion around the concept of pre-referral treatment, and whether the single dose treatment represents a ‘cure’ or not, can further blur the justification for referral [13]. Misunderstandings over the rationale for referral are dangerous both in locations close to hospitals where referral may be seen as less urgent due to easier access to referral facilities, as well as remote locations where delayed onset of referral could have serious implications.

While the literature suggests that caregiver recollection and understanding of referral advice has an important bearing on likelihood to complete referral [10], the actual content of referral messages provided by CHWs and how these are interpreted by caregivers, has been given less attention. Studies have suggested that even where CHWs convey referral messages correctly according to protocol, caregiver interpretation may deviate from the intended meaning of messages as they may process the information based on previous understandings of the subject [14]. That caregivers may be reluctant to admit misunderstanding information they receive from health care providers could also reduce efforts to seek clarification [15]. How CHWs communicate the concept of ‘pre-referral treatment’, how caregivers understand and contextualise the idea of pre-referral treatment, and subsequently use that information in decision-making around further care seeking, may all be important in influencing completion of referral.

A qualitative study was undertaken in western Uganda to explore how community understanding of the concept of pre-referral treatment impacted on referral related decision-making following the provision of RA. In Uganda, RA was introduced into the National Malaria Control Policy in 2011 as pre-referral treatment for all suspected severe malaria cases at lower level health centres (HCII and HCIIIs), as well as at the community level under the Integrated Community Case Management (ICCM) programme. In communities, CHWs are able to prescribe RA to any child aged four to 59 months presenting with a danger sign (including inability to breastfeed or drink, extreme sleepiness or unconsciousness, convulsions, excessive vomiting or prolonged fever for at least seven days) and following a positive malaria diagnosis from a rapid diagnostic test (RDT). At the time of data collection, CHWs were trained in the ICCM programme according to the ‘ICCM Facilitator’s Guide: Caring for Newborns and Children in the Community’ and were given the ‘ICCM sick child job aid’ to provide on-hand practical guidance to CHWs when interacting with their patients. Under this curriculum, CHWs were taught to introduce RA as ‘first aid’ so as to emphasise the need to seek further treatment. According to ICCM guidelines, after inserting the suppository the CHWs are to complete a referral form and refer the patient with some urgency to the nearest health centre with specific instruction to adhere to the referral even if the child improves on the way. To complete the referral process, caregivers are also encouraged to return the referral feedback from health centres to their CHW.

Three key sub-questions informed the study scope and line of enquiry:

1. What is the scope of message content provided by CHWs to caregivers with regards to the need, and reasons, for rapid transfer to a hospital after the administration of one dose of RA?
2. How do caregivers process and interpret the information provided by CHWs, what influences this interpretation or understanding, and what impact does this have on subsequent caregiver decision-making about referral?
3. What do caregivers in this context need to know in order for them to make an informed decision to rapidly complete referral following administration of one dose of RA?

These insights were expected to be important for optimising the uptake and use of RA in appropriate settings. The information generated from the study could be used to inform training curricula, CHW job aid content, supportive supervision guidance, as well as health education strategies.

## Methods

### Study site

The study was conducted in Hoima district in mid-western Uganda, which at the time of data collection had an estimated population of 600,000 people residing in 507 villages across 13 sub-counties. In February and March 2010, a total of 1059 CHWs (known as Village Health Team members/VHTs, in Uganda, but referred to as CHWs throughout this article) were trained and equipped in delivering ICCM. Hoima is mainly inhabited by the Banyoro people, with Runyoro the main language spoken. The district is predominantly rural, with smallholder subsistence farming the main economic activity. There are 42 public and 11 private health facilities in the district, with 77% of the population residing within 5 km from a health facility, according to national estimates. Confirmed malaria prevalence among children 0–59 months in the mid-western region remains quite high at 17.6% (same result according to both RDT and microscopy), though is lower than the national average at 31.7% and 20.0%, confirmed by RDT and microscopy respectively [16].

### Sampling and data collection

Selection of specific communities was guided by the Ministry of Health ICCM database which aggregates CHW register data including confirmed malaria cases, suspected severe malaria cases, RA administration and cases referred. Villages were sampled if they had seen at least one case of RA administration within the previous three months (the period from October to December 2012) and if they were located at least 5 km away from a health facility to which patients would be referred, as it is these communities for which pre-referral treatment is ideally intended. The sampling was done according to sub-county and parish administrative units which reflect health facility catchment areas and the coordination of CHWs. In total, 114 cases of suspected severe malaria among children under five had been given RA in this period, across 30 villages located at least 5 km from a health facility. These cases of RA administration were among an approximately equal number of males and females, mostly between the ages of 24 and 59 months. Specific demographic information of the patients or details of the episodes including symptoms of severe malaria were not extractable from the aggregated data in the ICCM database.

The first phase of data collection in the 30 villages identified was in January 2013 and involved series of in-depth narrative interviews with the CHW in the village who had administered RA the most recently (so as to aid recall), and the associated caregiver of that case. Narrative interviewing is a non-directive and in-depth research technique that encourages and stimulates an interviewee to tell a story about a significant event in their life and social context; it facilitates the reconstruction of social events from the perspective of the informant as directly as possible [17]. The narrative interview tool was a semi-structured interview guide organised around four main phases of a narrative interview as developed by Bauer (1996); initialisation (formulating initial topic for narration, using visual aids), main narration (no interruptions, only non-verbal encouragement to continue telling the story), questioning phase (focused on ‘what happened then?’ exmanent into immanent questions) and small talk (stop recording, memory protocol immediately afterwards) [17].

The transcribed stories from the narrative interviews were also used to develop context specific vignettes for discussion in a series of focus group discussions (FGDs) with caregivers of children under five and CHWs, as well as some women representatives, with the aim of gathering broader community opinion. The vignettes included both stories of adherence and non-adherence so as to solicit views on a range of scenarios reflecting different interpretations of the purpose of RA and referral advice given. It was hoped that by focusing discussion on context relevant case scenarios and away from direct personal experience, participants would share their opinions more candidly. FGD participants were identified purposively in collaboration with district and parish leaders with the aim of including caregivers and CHWs with direct experience of RA during the previous six months (the inclusion period for this data collection phase was raised to enable a larger number of eligible CHWs), as well as those with no recent experience. The sites sampled were different from those included for the narrative interviews so as to avoid contamination bias and to protect the identity of narrative interview respondents (names were also changed in the vignettes). Effort was also made to include a mix of ages. Women representatives, who are sometimes also CHWs, were included given their role as elected leaders in the community representing women on issues relating to health and so were considered well placed to boost the caregiver perspective. FGDs with caregivers were conducted separately according to gender, though males and females were combined for the CHW and women representative FGDs. Each FGD had a maximum of ten participants. This second phase of data collection took place in June and July 2013.

In-depth interviews (IDIs) with traditional healers were also conducted in the second phase of data collection with the aim of exploring their role in providing treatments rectally, which may have influenced caregivers’ understanding and interpretation of treatments delivered this way. Traditionally, herbal suppositories have been used in this area for epilepsy and to stop convulsions and it was hypothesised that this knowledge may have been transferred to RA, possibly with an expectation that the drug will have a curative effect. Traditional healers were identified and accessed through the local branch of the Uganda National Council for Traditional Healers and Herbalists Association. Participant selection was purposive and done in collaboration with the association’s leaders, with the aim of targeting practitioners with recent practical experience in treating children’s illnesses and to enable a range in terms of age and sex.

The topic guides for the narrative interviews, FGDs and IDIs were all piloted within a village considered typical for Hoima district but which was not targeted for data collection; subsequent minor adjustments were made. [See Additional file 1: Set of study interview guides.] Four field researchers were trained and recruited; two male and two female, all experienced in health-orientated qualitative research, familiar with the local socio-cultural setting and fluent in Runyoro. All interviews were audio-recorded, with notes written for back-up and verbatim transcription done on the same day as data collection so as to promote data validity and to enhance recollection and consideration of interview context. Transcriptions were written in Runyoro and translated later into English, with samples back-translated to check translation quality. Debrief meetings were held on a daily basis to review the scope and quality of data collected and to determine the level of data saturation.

### Data analysis

The analysis followed a meaning-based approach, whereby a coding sheet was initially developed based on the scope of data to be collected with emergent themes progressively added as the data were analysed. For the narrative interviews, the stories from the CHWs and corresponding caregivers were analysed as a series, enabling a review of consistency in responses and a data validity check. All coding was done in Word (Microsoft).

### Ethical considerations

The study protocol, data collection tools and consent protocols were subjected to ethical review and approval by the Uganda National Council for Science and Technology (no. HS1009). Informed consent (written and oral) was sought and obtained from all interview and focus group participants. The consent form included information on the broad aims of the study, confidentiality and respondent rights. No interviewees refused to participate in the study.

## Results

### Study participants

A total of 30 series of caregiver/CHW interviews were conducted across 12 sub-counties (a ‘division’ relates to an urban sub-county), involving 60 narrative interviews, 30 with CHWs and 30 with caregivers. A total of 19 FGDs were conducted with caregivers in 19 villages across 9 sub-counties, and 12 FGDs with CHWs and women representatives in 12 parishes across 12 sub-counties. A total of 20 traditional healers were interviewed. Details of the study participants are provided in Table 1.

**Table 1:** Study participants

Data collection method	Participants	Across no. of sub-counties
*Phase 1*		
Narrative interviews	30 interview series, comprising 30 CHWs (17m, 13f) and 30 caregivers (5m, 25f)	12
*Phase 2*		
Focus group discussions	19 FGDs with caregivers (7m, 10f, 2 mixed; total of 182 participants)	9
	12 FGDs with CHWs and women representatives (all mixed sex; total of 115 participants)	12
In-depth interviews	20 traditional healers (10m, 10f)	10
Total	60 narrative interviews, 31 FGDs, 20 semi-structured interviews. A total of 377 participants	

### Scope of message content by CHWs

The CHWs were broadly aware of the scope of essential information to be given to caregivers on prescribing RA, including explanation about rectal administration and the urgency for referral, as specified in the CHW ‘ICCM sick child job aid’ and emphasised during ICCM training. However, the breadth of content shared with a given caregiver appeared to vary depending on the time of the day or night the child was seen, the condition of the child and perceived urgency with which the CHW needed to act to save the child; in cases perceived as particularly severe, the rush to administer RA and support referral tended to reduce the scope of information shared. With cases perceived as less urgent, CHWs generally reported waiting for some time (i.e. up to 20 minutes) to see if the child’s condition stabilised and using this time to communicate more with the caregiver about the symptoms of severe malaria, the role of RA and importance of referral, and to respond to caregiver questions.

Caregivers tended to be familiar with artemisinin combination therapy (ACT) for a child that tested positive for malaria and in most cases came expecting the CHW to test the child and prescribe ‘Coartem’ (artemether-lumefantrine, the first line treatment in Uganda). Few knew about RA and for the majority of caregivers interviewed in the first phase, this was their first experience of RA.

Both CHWs and caregivers frequently reported that the role of RA was communicated as a “*first aid*” and the need to seek further treatment appeared to be broadly understood among most caregivers. Other reasons commonly mentioned by CHWs for prescribing RA were that it would stop convulsions, it should be given when a child cannot swallow or is vomiting (and therefore oral medicine can be hard to digest), and that it would help improve or stabilise the child’s condition; these all reflect guidance included in the job aid.

*I told her that I don’t have medicine for severe malaria but that I would give her some drugs to act as first aid so that she proceeds to hospital.* (Interview, CHW, Busiisi division).

*The CHW gave the child first aid by administering those capsules and he told us to go immediately to the health facility without fail. …He did not tell me why he had administered the drug but just told me it was to help me get to the health facility before the child’s condition gets worse. …He told me had given that treatment to the child to help me move the child to Mparangasi [health centre].* (Interview, Caregiver, Kyabigambire sub-county).

However, nine caregivers reported that RA would help the child get better. While CHWs did not report conveying this message specifically, a number of CHWs (17) reported explaining that “*RA works like other anti-malarials*”, with the aim of reassuring caregivers by relating RA to a more familiar treatment. While many CHWs reportedly also emphasised that further treatment was needed to supplement RA, the comparison with anti-malarial treatment may have contributed to misguided assumptions among caregivers of the role of RA as a comprehensive treatment for malaria in itself.

*When they bring children with malaria with a danger sign (severe malaria) and you insert rectal artesunate in the rectum, they take it that you have given the child a dose like Coartem. To them when you insert rectal artesunate they look at it as a cure for that illness and yet it is first aid that we give to help the caregiver move the child to a health facility for treatment. We have explained this to caregivers but they have not taken it seriously.* (Interview, CHW, Buhimba sub-county).

The important point that RA was not a full treatment for severe malaria was in many cases more implied by CHWs rather than specifically emphasised through statements such as “*I don’t have the medicine”, “I am not trained to treat this type of malaria*”, “*RA will help the child to get better and to stabilise”,* or “*I cannot/ will not give any further treatment so you will need to go to the health facility*”.

A number of CHWs emphasised what to do “*in case the situation worsened…”* which could have implied the need to monitor the child and decide on subsequent course of action to a change in the child’s condition, rather than automatically completing referral. Many CHWs did emphasise however that even if the child’s condition improved following RA administration, the child should still be taken to a health centre. Most CHWs and caregivers reported that a sense of urgency was communicated through emphasis on the severity of the illness and by pointing out the risks associated with delaying access of further treatment. Many CHWs also underlined urgency by telling caregivers that they did not need to queue at the health centre, but proceed directly to a health worker to get the child attended to. In a few cases, CHWs also directly facilitated a prompt referral by escorting the caregiver to the health centre.

*After she inserted [the RA] the CHW told me to prepare without wasting time and I [should] take her [the child] to the health facility within 20–30 mins…* (Interview, Caregiver, Kitoba sub-county).

*I told her that she should take the child immediately to the health centre because the child had a danger sign and that I couldn’t treat her.* …*I told her not to wait because the child was in a critical condition.* (Interview, CHW, Kigorobya sub-county).

A small number of caregivers reported that they could not recall what the CHW had said to them due to the panic and anxiety they were experiencing after seeing their children convulse or in a critical condition.

### How caregivers process and interpret the messages and impact on referral decision-making

Most caregivers appeared confident in the advice of the CHW. It was broadly understood that the CHWs were trained to handle particular conditions and not others, and that their care would “*stop somewhere”* from where more qualified professionals could take over. Those caregivers who conveyed this tended to understand more clearly that RA was a temporary measure and was not a curative treatment. Caregivers who noted a familiarity with their CHW, particularly due to the CHW’s previous care of their children, appeared more likely to accept referral advice.

*I think the CHW had given treatment to stop convulsions but the real malaria was not treated, the reason he referred her to go and see a health worker. Health workers have different levels of training and because of that, the CHW works to the level he can stop, that is why he referred Joyce to the health facility to see more qualified health workers.* (FGD, Caregivers, Buhanika sub-county).

Previous experience of RA among caregivers, while generally limited, was found to positively influence their response to RA and increase likelihood of referral. Where caregivers were personally familiar with the treatment and how it worked, they reportedly requested it, noting its effectiveness.

*That tablet is very good and it is given to children who have too much malaria that is characterised by convulsions. So when the CHWs insert that tablet, the child improves and convulsions stop. In fact that tablet helps the child to gain energy.* Asked if the capsule can cure malaria by itself, the respondent replied, *No, I don’t think that tablet alone can cure malaria because after inserting it, the CHW writes a referral form so that I take the child to hospital for further treatment. I think that tablet only helps the child to regain consciousness and it is after getting treatment from the hospital that the child cures.* (Interview, Caregiver, Kyabigambire sub-county).

Seven of the 32 caregivers did not complete referral post administration of RA, which appeared to result from a combination of factors. As perhaps expected, adherence to referral advice appeared to be higher when the child’s symptoms were considered severe. Some caregivers were also unsure as to whether the administration of RA represented a comprehensive treatment for severe malaria, as is suggested above; just under a half were confident in stating that RA was not a complete cure and that further treatment would be necessary. The remainder tended to be unsure, commonly saying that it depended on the condition of the child. As expected, the caregivers who appeared confident that RA was not a cure for severe malaria seemed more likely to complete referral. Similarly, many of those who were uncertain about the purpose of RA either did not complete referral or reported they were never referred. Furthermore, previous experience with ACT (Coartem most commonly mentioned) as a treatment for uncomplicated malaria, as well as perhaps comparisons by CHWs with “*other anti-malarials*”, appeared to lead to misconceptions that RA was a comparative complete treatment for severe malaria, thus reducing likelihood to complete referral.

*I think this drug also works like Coartem because the moment it is inserted in the bottom, it helps to reduce on severe malaria.* (FGD, Caregivers, Kyabigambire sub-county).

In about one third of the cases, CHWs also reported prescribing Coartem to children suspected of severe malaria after giving them RA, contravening practice guidelines and likely raising confusion over the role of RA and need for referral; eight caregivers who did not complete referral were also given Coartem. CHWs were seemingly more inclined to give Coartem if the caregiver appeared reluctant or unlikely to complete referral.

*When she brought the child, I tested, administered RA and told her to take the child to the health facility the following day but the moment she saw the child playing, she concluded that the medicine had really worked and the child was now fine. But … with such children in that state, you add them Coartem because the child may convulse again.* (FGD, CHWs, Kigorobya sub-county).

When CHWs implied, or the caregiver had interpreted, a need to monitor the child’s condition post RA administration, this appeared to raise confusion around the purpose of RA and completion of referral was less likely.

*I told the grandmother that the RA tablets that I had inserted will help the child to feel better but emphasised that she should take the child to the hospital the following morning. I wrote for her a referral form …. So, we slept and in the morning the child was much better and the grandmother said “since the child is now better, why should I go to hospital? You really helped me”. I told her that the malaria might come back and attacks the child and she replied “eh, if the malaria is back, then I will take the child to hospital”.* (Interview, CHW, Buhimba sub-county).

*We women we fear convulsion so much, but when the child is eating, walking and playing, we don’t get scared so much and true we take our time like you can even first go to the garden, cook food, wash the child…However, when the child is convulsing you cannot do all that before seeking treatment.* (FGD, Caregivers, Bugambe sub-county).

Three caregivers interpreted referral advice to mean that the child was so badly off to the extent that the CHW had failed to manage the case, hence the referral. In all these cases, caregivers thought the child could die and all promptly completed referral.

*I knew that the child’s condition was bad and that is why I was referred to the health centre. So I was a bit worried because usually I take my children to the CHW and the children get better but now that he had referred me, I was scared but the CHW told me that the child would be fine.* (Interview, Caregiver, Busiisi division).

It should be noted that failure to complete referral was only partially a result of caregivers’ interpretation of the information given by CHWs. Other factors mentioned by caregivers included distance to referral centres, lack of means of transport or money, being night time and so the health centre would not be open, and a general preference for seeking care from within the community. The need to consult husbands on the need for referral was also commonly raised for causing a delay in referral. Some traditional healers reported that caregivers sometimes seek treatment from them instead of completing referral to a health centre, often due to a lack of money. The data did not suggest an increased confidence by caregivers in any traditional over biomedical medication when severe malaria was suspected.

*There are those who bring children when they are very sick and when I ask, “did you go to the health facility?” someone will reply and say, … “I do not have money”. You also see the person that surely he or she has no money and you become sympathetic and you decide to give them herbs.* (Interview, Traditional healer, Kitoba sub-county).

In general, knowledge of, or experience with, traditional herbal rectal medications, appeared to positively contribute to the acceptability of RA. Herbal medicines have traditionally been used to treat both childhood and adult illnesses in Uganda, in particular for epilepsy, to stop convulsions, constipation or swelling of the rectum, and while the practice has reduced over time, it is culturally accepted. Modern medicines administered rectally such as rectal diazepam have also been in use. Although many of the study participants had not personally experienced rectally administered herbal (or modern) medications, most had heard about them and knew they were available.

*Our great grandparents used to tell us how they administered local medicine locally known as ‘entego’…they were using pumpkin leaves and this kind of medication was being given in case the child had constipation. … I also know of a disease locally called ‘omwoyo’ whereby in case a child is sick, they rub those herbs around the affected areas especially in the anus. …Omwoyo is more of the anus [protruding] out but it is treated by a local herb called ‘omununu’ which they smear or try to push in the anus.* (FGD, Caregivers, Kyabigambire sub-county).

*[Parents whose children were treated with ‘entego’] would appreciate it because they knew about entego that it is there and it works. …entego is a cultural practice among the Banyoro people. Even me, when I was born I found it there and even others who were born after, they found it there.* (Interview, Traditional healer, Kitoba, sub-county).

### What information would support urgent referral completion?

It was commonly suggested by caregivers that CHWs should give more emphasis to the need for urgent referral following administration of RA, whether the child’s condition changes or not. That RA is not a complete cure for severe malaria, nor a full course of treatment, was also raised by many as critical information to be clearly imparted to caregivers. The message that RA is a ‘first aid’ appeared to resonate well with caregivers and was highlighted for further emphasis.

*The CHW needs to tell the caregiver why she should go to the health facility and not just give a referral form assuming that the caregiver will just go. Such an act may not make sense to the caregiver especially after seeing the child improving.* (FGD, Caregivers, Kiziranfumbi sub-county).

*The CHW should have told the caregiver that “the treatment I have given is only to help you as you take the child to the health centre not the real treatment”. So you assure her that it is not the final treatment, “the best drug for the treatment is at the health centre”. … She should have explained to the caregiver that there is no extra medication given by CHWs for severe malaria.* (FGD, CHWs and Women Leaders, Kabwoya sub-county).

Many participants emphasised the importance of CHWs explaining the dangers of not completing referral, in particular the risk of death or more severe malaria infection. A number of caregivers mentioned the value in *“scaremongering”*.

Both CHWs and caregivers suggested that CHWs should also share more general information about RA, including what it is for and how it works, given it is still a relatively new drug. Specifically, it was suggested that explaining the approximate time span between RA administration and receipt of further medical attention (i.e. how long RA is effective for) and practical aspects such as whether the tablet will dissolve or needs to be removed, and what to do should the child defecate soon after administration, would be useful. Guidance on alternative action in case the referral centre is closed was also suggested. The importance of follow up by CHWs was also mentioned by a number of caregivers as a motivator for referral action. Some caregivers also indicated that CHWs should do more to encourage urgent referral such as telephoning health centres to notify them of a referred patient, the consistent provision of a referral note and where possible, direct accompanying of the caregiver to the health centre.

## Discussion

The study suggests a clear link between caregiver understanding of the basis for and need to complete referral post administration of RA, and adherence to referral advice. That caregivers who appeared more confident that RA was not a cure for severe malaria seemed more likely to complete referral is an illustration of this.

The importance of key information to be imparted by CHWs on RA alongside its administration seems to be critical in encouraging appropriate understanding of the role of RA as a pre-referral treatment. CHW generally adhered to the essential information on RA provided through the ICCM training when communicating key messages to caregivers, though the study did not aim to evaluate this comprehensively. It is worth noting however that the information on RA administration in both the ‘ICCM Facilitator’s Guide: Caring for Newborns and Children in the Community’ and the ‘ICCM sick child job aid’ is limited and almost completely clinically orientated; the former focused on when to give RA in the context of danger signs and how to give according to age groupings and dosages, while the latter provides brief practical support only with no suggested accompanying key messages to impart to caregivers. Role play approaches and practice sessions did feature during the ICCM training though again were focused on the process for insertion of RA and decision making on its provision considering danger signs and age of the child. There was no specific guidance provided in either the training or job aid on what the CHWs should tell the caregivers alongside the administration of RA.

Perhaps arising in part from this lack of clear guidance on messages, the scope of information given to caregivers by CHWs did vary considerably, affected by the time of day or night the child was seen, the presenting condition of the child, and the perceived urgency with which the CHW needed to act; in many cases, more information tended to be given to caregivers of children whose cases were perceived as less severe. It is implied that CHWs tend to prioritise key messages to share, balanced alongside the focus on clinical action and the perceived urgency of referral.

The indicated level of completed referrals to a health centre also corresponds well with another study which found that a quarter of the caregivers did not comply with referral advice post administration of pre-referral treatment [10]. Adherence to referral advice appeared to be boosted by an understanding that RA acted as a ‘first aid’ which relieved fever and convulsions temporarily (thereby enabling the chance to reach the health centre for further treatment), confidence in the advice of the CHW arising from previous interactions, previous experience of RA, the severity of symptoms, as well as experience or familiarity with traditional herbal rectal medications. The link between severity of symptoms and probability of adherence to referral advice has also been found elsewhere [10, 12]; in one setting, the odds of adherence for children with altered consciousness and/or convulsions was three times greater than for children with other symptoms. Other studies also reported a positive contribution of previous experience of traditional herbal rectal medications on the acceptability of RA, including in Uganda [4], Tanzania [8], Papua New Guinea [5] and Zambia [9]. While it was hypothesised that caregivers’ knowledge about rectally administered herbal medications, which aim to comprehensively treat some illnesses, may be transferred to RA in that some caregivers may expect RA to be curative, this study found no indication that this was the case.

Achieving the appropriate balance between conveying a sense of ‘urgency’ for referral, post administration of RA, alongside the message that ‘time is bought’ appeared to be hard to strike due to the potential for misguided assumptions and misinterpretations. That RA is not a full treatment for severe malaria appeared to be emphasised more by CHWs than caregivers suggesting a communication gap; there is a need to be very specific and clear when delivering this message. The role of ‘pre-referral treatment’ should be well defined and articulated to all players in the referral process – the use of the word ‘treatment’ itself can create confusion given curative connotations and sensitive differences can be lost in translation. It also seemed likely that comparison with the more familiar anti-malarial ‘treatment’ contributed to misguided assumptions among caregivers of the role of RA as a comprehensive treatment for malaria in itself; comparisons focused on differences rather than similarities may support better understanding of the nature of pre-referral treatment.

It was seemingly understood by some caregivers, with understandable logic, that if RA causes the child’s convulsions to stop, this indicates a safer state of health and means further treatment may be unnecessary. A number of CHWs emphasised what to do “*in case the situation worsened…”* which appeared to imply the need to monitor the child and decide on a course of action depending on a change in the child’s condition, rather than automatically completing referral. Improvements in the child’s condition following RA administration, indicating to some that the child was getting better and thus reducing the likelihood of referral completion, has been found in other settings [10]. There is a clear need for more comprehensive information and practical guidance to enable caregivers to effectively respond to a range of circumstances post RA administration; caregivers have an active role to play in the urgent completion of referral and need to be informed consumers. The important link between the level of information received by the caregiver or patient and the trust generated in relation to the proposed treatment plan has been raised elsewhere [18].

### Implications

To boost the effective administration of RA, there is a need to both improve the performance of CHWs and encourage caregivers to complete referral.

For CHW performance improvement, it is important that the development and adaption of training materials adopt more comprehensive guidance to RA administration beyond clinical recommendations and practicalities of provision, to include key messages to impart to caregivers, likely caregiver questions or areas of confusion, and inter-personal communication skills and approaches for CHWs. An emphasis on interactive training sessions and the adoption of adult learning methods is also recommended. The ICCM programme is broad with considerable guidance required for the correct identification of danger signs, treatment, pre-referral treatment, related advice and home care for malaria, pneumonia and diarrhea, as well as routine care for newborns; follow-up, hands-on refresher training could focus on each aspect of ICCM separately to give each sufficient attention. It is important that CHWs understand well the concept of pre-referral treatment, as well as how to manage cases when caregivers refuse referral. There may also be a need to intensify both individual and group support supervision to enable direct review of CHW performance in their own setting as well as the sharing of experiences among CHWs.

A good communication strategy is also needed to encourage referral adherence [19, 20] and specific, clear guidance should be provided on key messages to share, as well as the prompting of caregivers to raise any concerns around being referred, with an overall emphasis on interaction and ‘counselling’ over ‘telling’. Given the need to prioritise key information so as to retain simplicity and to avoid confusion, job aids are usually focused on clinical guidance with less emphasis on key messages to impart to or key points to discuss with caregivers. Based on the findings of this study, Table 2 suggests some key information for CHWs to emphasise and discuss with caregivers alongside the administration of RA, to be adapted as appropriate to suit local contexts. In particular, that RA provides a temporary relief prior to facility-based treatment, the differentiation (rather than similarities) between RA and ACT in terms of curative aims, the importance of referral whether or not a change is seen in the child’s condition and the risks of not completing referral should all be given more focus. Careful attention should be given to the translation of materials into local languages to enable accurate messaging, particularly around the concept of ‘pre-referral treatment’; it might be preferable to use the word ‘medicine’ instead of ‘treatment’. CHW-led community information sessions may also be valuable when introducing new or novel medicines to community health service delivery so as to raise general awareness and to address potential misconceptions, and in this case, to improve understanding of the importance of referral adherence following pre-referral treatment. Such sessions could also raise general awareness of the scope of CHW services. More priority should also be given by CHWs to promoting completion of referral. It should be noted however that CHWs are volunteers and as such, the balance between engaging them and overburdening them with frequent activities should be considered.

**Table 2:** Scope of suggested key messages for CHWs to emphasise and discuss with caregivers when administering RA so as to boost referral adherence

CHW key messages	
• Purpose of RA is a pre-referral treatment that provides temporary relief to the child and enables the caregiver some time to seek urgent, further treatment from the health centre – i.e. it acts like a ‘first aid’	
• RA is not a cure for severe malaria and works in a different way to anti-malaria treatment (ACT i.e. Coartem). CHWs cannot give the recommended treatment for severe malaria – this must be given at a health facility	
• Having been given RA, the child may seem a little better but this improvement is temporary and the child’s condition is still critical – symptoms may return. Referral remains urgent	
• Beyond urgent referral, caregivers need not take any further action once the RA has been inserted – the tablet will dissolve, it does not need to be removed and defecation will not stop it from working	

### Limitations

This study’s specific research question is intertwined in a complex web of issues related to care-seeking, local explanatory models of childhood illness, feelings about referral and the overall health system, perceptions and experiences of the care available from either CHWs or health facilities; this created a challenge in maintaining the study’s focus throughout data collection and the relevance of wider issues must be considered.

Gathering data retrospectively about an event, and in particular communication related to that event, can be challenging and the data may have been affected by recall bias. This effect was minimised by limiting the period of eligibility for in-depth inquiry; only caregivers and CHWs who had been involved with a case requiring RA administration within the preceding three months could participate.

It is possible that caregivers who were referred without completing referral did not admit this during interviews, instead reporting that they were not referred or had been told by the CHW to go the health centre only if the child’s condition did not improve. The potential effect of social desirability bias must be considered in data interpretation, though the most likely reasons for not completing referral were considered to have emerged during data analysis.

## Conclusions

Caregiver interpretation of CHW advice relating to pre-referral treatment was found to influence their decisions to promptly complete referral. A clear link was implied between caregiver understanding of the basis for and need to complete referral post administration of RA and adherence to referral advice. While information shared by CHWs related closely to guidance provided in the job aid, scope and consistency varied depending on the perceived urgency with which the CHW needed to act and the time of day or night the child was seen. Around a quarter of caregivers did not complete referral post administration of RA which is consistent with findings in other settings. Caregivers seemed more likely to adhere to referral advice if they perceived their child’s condition to be severe. Previous caregiver experience and CHW comparisons with ACT as a treatment for uncomplicated malaria appeared to raise misperceptions that RA is a complete treatment for severe malaria, thus reducing likelihood to complete referral. Caregiver interpretation of the need to monitor the child and, in some cases, the prescription of ACT post RA administration also confused the need for referral. A good communication strategy is needed to boost referral adherence and careful attention should be given to the translation of messages relating to ‘pre-referral treatment’. In particular, that RA provides a temporary relief prior to facility-based treatment (rather than being a full treatment for severe malaria), the differentiation (rather than similarities) between RA and ACT in terms of curative aims, the importance of referral whether or not a change is seen in the child’s condition and the risks of not completing referral should all be given more focus. Social behaviour change communication, CHW training and refresher training activities and intensified CHW support supervision could help promote these messages.

## Additional file

Additional file 1: Set of interview guides for the study, ‘Community understanding of the concept of pre-referral treatment and how this impacts on referral related decision-making following the provision of rectal artesunate: A qualitative study in western Uganda’. Interview guides file includes: 1. Narrative interview guide for Village Health Team members (Uganda specific name for CHWs); 2. Narrative interview guide for caregivers; 3. Focus group discussion guide for Village Health Team members and women representatives; 4. Focus group discussion guide for caregivers; 5. In-depth interview guide for traditional healers. (DOCX 31 kb)

## Abbreviations

ACT: Artemisinin-based combination therapy
CHW: Community health worker
FGD: Focus group discussion
HC: Health centre
ICCM: Integrated community case management
IDI: In-depth interview
RA: Rectal artesunate
RDT: Rapid diagnostic test
WHO: World Health Organization
