# Supplementary material for: Community understanding of the concept of pre-referral treatment and how this impacts on referral related decision-making following the provision of rectal artesunate: a qualitative study in western Uganda
Source: BMC Health Serv Res. 2018 Jun 19;18:470. doi: 10.1186/s12913-018-3209-4 (PMC6006771; doi:10.1186/s12913-018-3209-4)
Supplement: Supplementary file 1 — Set of interview guides for the study, ‘Community understanding of the concept of pre-referral treatment and how this impacts on referral related decision-making following the provision of rectal artesunate: A qualitative study in western Uganda’. Interview guides file includes: 1. Narrative interview guide for Village Health Team members (Uganda specific name for CHWs); 2. Narrative interview guide for caregivers; 3. Focus group discussion guide for Village Health Team members and women representatives; 4. Focus group discussion guide for caregivers; 5. In-depth interview guide for traditional healers. (DOCX 31 kb) [file 12913_2018_3209_MOESM1_ESM.docx]

**Additional file 1**

**Set of interview guides for the study, ‘Community understanding of the concept of pre-referral treatment and how this impacts on referral related decision-making following the provision of rectal artesunate: A qualitative study in western Uganda’.**

This set includes:

1. Narrative interview guide for Village Health Team members (Uganda specific name for CHWs)
2. Narrative interview guide for caregivers
3. Focus group discussion guide for Village Health Team members and women representatives
4. Focus group discussion guide for caregivers
5. In-depth interview guide for traditional healers
6. **Topic guide for narrative interviews: Village Health Team (VHT) members**

Eligibility: Have administered rectal artesunate to a child with suspected severe malaria during the previous three months.

**Following receipt of informed consent, record the following information:**

1. Name of interviewer:
2. Name of note-taker:
3. Date of interview:
4. District:
5. Sub-county:
6. Village:
7. Name of LC1 Chairperson:
8. Age of VHT:
9. Sex of VHT:
10. Name of child who most received RA by the VHT:
11. Sex of child who most received RA by the VHT:
12. Age of the child who most received RA by the VHT:
13. Date when child received treatment with RA:
14. **Initialisation phase**

Q1: Please describe the effect of malaria in your community. Do you see many children under 5 with severe malaria? How many cases of severe malaria have been brought to you during the last 1 month? 3 months?

Q2: How do you find using rectal artesunate on children with severe malaria?

(**Probes**: Do you always have stock?)

1. **Main narration phase**

**(Interviewer: At this stage you will ask the VHT to tell you the story about when they administered rectal artesunate to a recent patient. Do not ask questions or interrupt the story.)**

**(Confirm: *You have said you administered rectal artesunate to a child in the past 3 months, right?*)** Please tell me about what happened the last time you administered rectal artesunate to a child. It’s better to tell me in details, in form of a story. You are free to just tell me what you remember on that day, there is no right or wrong response here. We are simply interested in hearing your story, in your own words. You can start with the time when the caregiver first came to you with the child showing signs of severe malaria.

1. **Questioning phase**

**(Once the VHT has finished telling the story, you can now ask probing questions about any parts of the story that were not clear or where you need more information. The questions below may or may not be asked, or you can come up with other follow-up questions – it is important that they are linked to the story just told though. Probe questions should be mostly about events - *e.g. what happened next?* – as well as reflections/ feelings - so as to access the detail of the story.)**

Q1: (**Probes:** Start these by referring to the story, e.g. *“you mentioned that …. What happened before that/after that?”*)

- What symptoms did the child have?
- What did you say to the caregiver before giving rectal artesunate?
- How did the caregiver react to the idea of giving their child rectal artesunate?
- What did you say to the caregiver after administering rectal artesunate? How did they respond? Did they ask any questions?
- What happened next? Did the caregiver do as you advised?
- If the caregiver took their child to the health facility, how quickly did they go?
- Why do you think the caregiver acted as they did?
- Was anybody else involved in the caregiver’s decision of whether to take their child to the health facility so as to complete the referral?

Q2: Thinking about what happened, is there anything you would have done differently with regards to the child?

(**Probes:** Is there anything you think you should have said to the caregiver that you did not say? Why would that be important?

If you saw another caregiver with a child with severe malaria today, would you respond in the same way? Why/why not? Would you say exactly the same things or different things or anything else?

What else or what different things would you say to the caregiver to encourage them to take up the referral?)

Q3: Do most caregivers whose children are given rectal artesunate by you complete the referral or not?

(**Probes:** What do you think are the reasons for this?)

Q4: What do people in your community generally say about rectal artesunate?

1. **Small talk**

Q1: Is there anything else you would like to say, any additional information, or something you have forgotten to mention?

Q2: Do you have any questions to ask me? Or any thoughts, recommendations or suggestions on today’s topic?

**Thank the respondent for his/her time.**

**Make notes about how the discussion went; what are your impressions? Did the informant talk freely? Did they seem to remember what happened well?**

1. **Topic guide for narrative interviews: Caregivers**

Eligibility: Caregivers’ child received rectal artesunate in the previous three months.

**Following receipt of informed consent, record the following information:**

1. Name of interviewer:
2. Name of note taker:
3. Date of interview:
4. District:
5. Sub-county:
6. Village:
7. Name of LC1 Chairperson:
8. Age of caregiver:
9. Sex of caregiver:
10. Number of people in household:
11. Number of children under 5 in household:
12. Name of VHT who administered RA to caregiver’s child:
13. Sex of VHT who administered RA to caregiver’s child:
14. Age of the child:
15. Sex of the child:
16. Date when child received treatment with RA:
17. **Initialisation phase**

Q1: ***Show picture of a VHT administering rectal artesunate to a child, and ask****:* What do you see in the picture? What do you think is going on?

Q2: Have you heard about rectal artesunate (the medicine/tablet that is inserted into the child’s bottom/anus/rectum)?

Q3: What do you know about rectal artesunate?

1. **Main narration phase**

**(At this stage you will ask the caregiver to tell you the story of when their child recently received rectal artesunate from a VHT when they had suspected severe malaria. Do not ask questions or interrupt the story.)**

**(Confirm: *You have said your child/children have been treated with rectal artesunate, right? Was it you yourself? Did you go alone?*)** Please tell me about what happened the last time your child fell sick and was given this medication by inserting it in the rectum. It’s better to tell me in details, in form of a story. You are free to just tell me what you remember, there is no right or wrong here. We are simply interested in hearing your story, in your own words. You can start with the time when you first noticed the illness until after the child was provided with rectal artesunate, and what happened thereafter.

1. **Questioning phase**

**(Once the caregiver has finished telling the story, you can now ask probing questions about any parts of the story that were not clear or where you need more information. The questions below may or may not be asked, or you can come up with other follow-up questions – it is important that they are linked to the story just told though. Probe questions should be mostly about events - *e.g. what happened next?* – as well as reflections/ feelings - so as to access the detail of the story.)**

Q1: (**Probes:** Start these by referring to the story, e.g. *“You mentioned that …. What happened before that/after that?”*)

- What signs or symptoms did the child have?
- How long after you thought your child started to get sick did you go to the VHT?
- What did the VHT do first?
- What did he say to you before giving that medication?
- What did the VHT say after inserting the medication?
- What other information did s/he provide to you?
- What did you think of the VHT’s advice?

**(Ask Qs 2-6 only if the child was actually referred from the VHT.)**

Q2: How did you feel when the VHT told you to take the child for further treatment at the health facility?

(**Probes:** Why did you feel that way?)

Q3: What did you do then? Did you go to the health facility?

(**Probes:** What are the reasons you followed/did not follow the VHT’s advice? Where did you go?

**If went to the health facility**: How did you get to the facility? Did you get there quickly?)

Q4: How did the information given by the VHT influence you to take the decision/ actions you took?

(**Probes:** What else did you have to think about to take a decision to go or not to go to the health facility?)

Q5: Did you talk to anybody before taking the decision to go/not to go?

(**Probes:** Did anyone influence your decision about whether or not to go? How?)

Q6: (**Only for those who were referred and did not go the health facility immediately or at all).** What would have made you immediately take your child for further treatment at the health facility as advised by the VHT?

**(Ask Q7 if the child was not referred by the VHT.)**

Q7: Why do you think the VHT did not refer you to the health facility?

(**Probes:** Would you have preferred to be referred to the health facility after the treatment that the VHT gave your child? Why?)

**(Ask Q8 if the child completed referral.)**

Q8: Were you attended to at the health facility? What did they do? How did your child respond to the care/ treatment they were given?

**(Ask all.)**

Q9: Is there more information that you would have liked to receive from the VHT? What would this be?

(**Probes:** Why do you think this would have been useful?)

Q10: Did you use any other medications on your child during that illness episode?

(**Probes:** If yes, what medications? How was your child after they were given?)

Q11: If you went through the same experience again, would you do the same as you did last time? Why/why not?

Q12: Are there any traditional medications used in your community (for any illness) that are administered through the bottom/rectum?

(**Probes**: Have they ever been used on any of your children?

**If yes:** Can you tell me more about that? What illnesses did they have? Who gave the medication? Was the medication used alone or with other medications? Do you think it helped your child?)

1. **Small talk**

Q1: Is there anything else you would like to say, any additional information, or something you have forgotten to mention?

Q2: Do you have any questions to ask me? Or any thoughts, recommendations or suggestions on today’s topic?

**Thank the respondent for his/her time.**

**Make notes about how the discussion went; what are your impressions? Did the informant talk freely? Did they seem to remember what happened well?**

1. **Topic guide for focus group discussions: Village Health Team (VHT) members and women representatives**

Eligibility: VHTs trained in the administration of rectal artesunate to children with suspected severe malaria, some of whom had recent experience in the administration of rectal artesunate to children under five (during the previous six months) and some who did not. Many elected women leaders are also VHTs; while women leaders who are not VHTs have not been trained in the clinical administration of rectal artesunate, they are likely to have useful insight into the surrounding issues.

**Following receipt of informed consent, record the following information:**

1. Name of interviewer:
2. Name of note taker:
3. Date of FGD:
4. District:
5. Sub-county:
6. Village:
7. Name of LC1 Chairperson:
8. Number of FGD participants:
9. Sex of FGD participants (m/f):

**Questions:**

Q1: Please describe the effect of malaria in your community. Do you see many children under 5 with severe malaria? How many cases of severe malaria have been brought to you during the last 3 months?

Q2: How can you tell if a child has severe malaria?

Q3: What do you do if you receive a suspected case of severe malaria? Please tell me the step-by-step process of what you usually do, with explanations as to why you do those things.

(**Probes: Explore rationale and detail of each step discussed if not given** i.e. Why give rectal artesunate? For what signs and symptoms? Why wait a few minutes? Why refer? etc.)

Q4: Do you have problems with stocks of rectal artesunate?

(**Probes:** Any stock-outs in the past six months? For how long?)

Q5: I am going to read for you a true story of a VHT called “Petero”, though this is not his real name. Listen carefully to the story, and then we will discuss it when I finish reading it.

**Read out the story at least twice, loud and clearly enough for all participants to hear.**

Last week, Petero, a male VHT in Karaaro village, received a case of a child aged 2 years who had a high temperature, was vomiting, and was not playing as they normally do. The mother of the child also reported that the boy had convulsed before she brought him. Indeed, the boy convulsed again when Petero was still checking him.

Petero did a rapid test and it was positive for malaria. He then administered 2 rectal artesunate suppositories in the baby’s bottom. Petero asked the mother to take the child home and only come back if the child does not get well.

**Questions about the story:**

1. What thoughts came to your mind as you listened to this story?
2. What information is missing in the story? What else would you have liked to know about this incident? Why do you need to know this?
3. What do you think about the VHT’s course of action?

(**Probe:** What do you think about the VHT’s advice to the mother to go home and come back only if the child does not get well?)

1. Is there anything else that the VHT needed to do?
2. If you were the VHT, would you have behaved in the same way or differently? Why?
3. What questions did the VHT need to ask the caregiver before administering rectal artesunate to the child?
4. Is there anything else you would like to say about this story and how the VHT responded to the patient?

Q6: I am going to read for you another true story about a VHT from a different village. In the story she is called “Yunia”, though that is not her real name. Listen carefully to the story, and then we will also discuss it when I finish reading it.

**Read out the story at least twice, loud and clearly enough for all participants to hear.**

Yunia, a female VHT in another village, received a case of a child aged 3 years who had a high temperature, was vomiting, and was also playing well with others as she usually did. The child had convulsed twice and did so a third time as soon as they arrived at Yunia’s home.

Yunia administered rectal artesunate to the child’s rectum. She then did an RDT test for malaria and found it was positive. Yunia wrote a referral note and referred the caregiver to the nearby health centre. The mother left Yunia’s home but did not go to the health centre.

**Questions about the story:**

1. What thoughts came to your mind as you listened to this story?
2. What do you think about Yunia’s management of the patient? Would you have done anything differently?
3. What do you think about Yunia’s advice to the mother to take the child to the health centre?
4. Why do you think the mother didn’t go?
5. What information should the VHT have provided to the mother after giving the child rectal artesunate?
6. What more information would have encouraged the mother to go to the health centre?

**Further questions:**

Q8: When do you take an RDT test, before or after administering rectal artesunate? Why?

(**Probes:** What do you do if the malaria test is negative?)

Q9: How soon does the child need to get to the health centre after having been treated with rectal artesunate? What are the reasons for this?

Q10: Are there circumstances when you do not refer after giving rectal artesunate? What are these circumstances?

Q11: What do you usually say to the caregiver when you are explaining the need to go to the health centre after the child has been given rectal artesunate? Why do you say this?

Q12: How do the caregivers usually respond? Do they sometimes ask you any questions? What questions do they ask?

(**Probes:** Do you think they understand you well?)

Q13: Do they usually go to the health centre when the child has been referred? Why/why not?

Q14: To which health centre do you usually refer caregivers after administering rectal artesunate?

(**Probes:** Is it a government or private facility? Do caregivers usually go to the facility you refer them to, or do they change? What are the reasons if they change?)

Q15: Who in the household usually makes the decision about the referral after getting RA, such as whether to go, where to go, and when to go?

(**Probes:** Are the husbands involved in the decision making? In case the husband is not at home, can a mother make this decision on their own without waiting for her husband? What role is played by other family members e.g. grandparents, mother-in laws in this decision making?)

Q16: What do you as VHTs do to support caregivers to complete the referral?

Q17: How do people in your community feel about giving medicines to children through their rectum?

(**Probes:** Do they have any concerns about this? Why/why not? Do you know of any traditional medications for children or adults that are administered through the rectum? If yes, what medications? For what diseases? How do they work?)

Q18: Is there anything else you would like to say, or any additional information you would like to share? Do you have any questions to ask me?

**Thank the participants for their time.**

**Note down any key impressions and observations about the discussion, e.g. on composition of participants, the flow of the discussion, issues that raised a lot of debate.**

1. **Topic guide for focus group discussions: Caregivers**

Eligibility: Caregivers of children under five, some of whom had recent experience in their child receiving rectal artesunate from a VHT (during the previous six months) and some who did not.

**Following receipt of informed consent, record the following information:**

1. Name of interviewer:
2. Name of the note-taker:
3. Date of FGD:
4. District:
5. Sub-county:
6. Village:
7. Name of LC1 Chairperson:
8. Number of FGD participants:
9. Sex of FGD participants (m/f):

**Questions:**

Q1: Please describe the effect of malaria in your community. Do children often fall sick with malaria? How about severe malaria?

(**Probe**: when a child is *convulsing, vomiting everything, has neck stiffness, abnormally sleepy and difficult to wake, unconscious, not able to drink or breastfeed)?*

Q2: Where do you seek treatment for malaria? Does this change if you think the child may have severe malaria?

Q3: How do the VHTs treat a child when they suspect they have severe malaria?

(**Probes:** Who in this group have heard about rectal artesunate? How many have seen it being administered? How does it work?)

Q4: I am going to read for you a true story of a mother called “Joyce”, though this is not her real name. Listen carefully to the story, and then we will discuss it when I finish reading it.

**Read out the story at least twice, loud and clearly enough for all participants to hear.**

Last week, Joyce, a 30 year old mother of four, returned from her garden at 12 noon and found her 2 year old daughter had a high temperature, was vomiting, and was not playing the way she usually does. She was also unable to feed or swallow anything. After a few minutes, as Joyce was still wondering what to do, the baby started convulsing.

Joyce rushed the baby to the VHT in her village. The VHT tested the baby and confirmed that she had severe malaria. The VHT treated the baby by inserting 2 rectal artesunate suppositories into the baby’s bottom. The VHT wrote a referral note and advised Joyce to take the baby straight away for further treatment at the government health centre, which is 2 miles away.

Joyce said she needed to go back home to prepare herself and she left. By the time Joyce reached home, the baby had improved, the temperature had reduced and she started to play again. Joyce did not take the child to the health centre.

**Questions about the story:**

1. What thoughts came to your mind as you listened to this story?
2. What do you think about the VHT’s advice to Joyce that she should take the baby to the health centre straight away after having been given rectal treatment?
3. What do you think of Joyce’s action of not going to the health centre?

(**Probes:** Why do you think she decided not to go the health facility? Was this the right choice? Why/why not?)

1. Should the VHT have given Joyce more information or explained anything more to her?

(**Probe:** What more information do you think would have encouraged Joyce to go to the health centre?)

1. Is there anything else that the VHT needed to do? If yes, what?
2. If you were in Joyce’s place, would you have behaved in the same way or differently? How?
3. What lessons do you learn from this story?
4. What do you think may have happened afterwards (to the baby)?

**Further questions:**

Q5: What purpose do you think this medicine, rectal artesunate serves? How does it help a child?

(**Probe**: What do you think about giving rectal artesunate to a child in the community rather than at a health centre?)

Q6: Do you think it is important for a child to be taken to the health centre after s/he has been given rectal artesunate by a VHT? Why/why not?

Q7: If you think it is important that the child goes to the health centre after receiving rectal artesunate, how soon do you think the child needs to get there? What are the reasons for this?

(**Probe:** Is it a matter of urgency to get to the health centre? Why?)

Q8: As a caregiver, if a VHT refers you to a health centre after administering rectal artesunate to your child, what else would you need to know?

(**Probes:** What questions would come to your mind? What would you want the VHT to tell you about? Dot the VHTs usually provide this information? Why/why not?)

Q9: Do caregivers in your community who are referred to the health centre by VHTs after their child has been given rectal artesunate usually go to the health centre? Why/why not?

(**Probe:** What are the reasons why some may not complete the referral?)

Q10: To which health centre do VHTs usually refer you to after administering rectal artesunate to your child?

(**Probes:** Is it a government or private facility? Do you usually go to the facility you are referred to, or do a different one? Why?)

Q11: Who in the household makes the decision about the referral, such as whether to go or not, where to go, when to go and so on?

(**Probes:** Are the husbands involved in the decision making? In case the husband is not at home, can a mother make this decision on their own without waiting for her husband? What role is played by other family members e.g. grandparents, mother-in laws in this decision making?)

Q12: What roles do husbands play in actually taking the child to the health centre?

(**Probes**: Who pays for it? Do they usually accompany you and the child? Do they help with transport?)

Q13: How do you feel about giving medicines to children through their rectum?

(**Probes:** Do you have any concerns about this? Why/why not? Do you know of any traditional medications for children or adults that are administered through the rectum? If yes, what medications? For what diseases? How do they work?)

Q14: Is there anything else you would like to say, or any additional information you would like to share? Do you have any questions to ask me?

**Thank the participants for their time.**

**Note down any key impressions and observations about the discussion, e.g. on composition of participants, the flow of the discussion, issues that raised a lot of debate.**

1. **Topic guide for in-depth interviews: Traditional healers**

Eligibility: As available, but with the aim of including those with recent practical experience in treating children’s illnesses.

**Following receipt of informed consent, record the following information:**

1. Name of interviewer:
2. Name of note taker:
3. Date of interview:
4. District:
5. Sub-county:
6. Village:
7. Name of LC1 Chairperson:
8. Age of traditional healer:
9. Sex of traditional healer (m/f):

**Questions:**

Q1: Please tell me about the kind of children’s diseases or infections that you treat.

Q2: Roughly how many children have you seen over the last month? What problems did they have?

Q3: Can you tell me about the medications you give to children. How are they given?

(**Probes:** Are they given orally, smeared, rectally, through bathing, inhalation etc.? Do you know about the procedure ‘*entego’* – can you tell me about it? How is that given? For what other illnesses can children be given rectal medicine? Do children who are treated with rectal medications such as ‘*entego*’ get cured just with this treatment, or do they have to be given other additional treatments? How do caregivers feel/react when their children are to be given rectal medications? Why do you think they react like that?)

Q4: Do you give any treatment through the rectum for adults’ diseases? If yes, for what diseases? Are people usually happy to receive this treatment? How do they react? Does it make them better?

Q5: Do you sometimes get children with very high temperatures, vomiting, failing to eat, convulsions – all these symptoms at the same time? Do you treat these? How?

(**Probes**: What sort of treatments, and how are they administered? What illnesses cause these symptoms? Do you think a child who has all the above symptoms could be having severe malaria? Do you treat malaria? Do you treat severe malaria? Why do you think caregivers with these symptoms come to you? At what stage do they normally come with these symptoms i.e. early after detecting symptoms, late when the child is very sick, after seeing the VHT or visiting the health centre etc.?)

Q6: Have you ever received a case of a sick child who has been treated by a VHT, but the illness has persisted?

(**Probes**: Have you ever received a child that had been treated with rectal artesunate? Why was the child brought to you? What did you do? How did the child respond?)

Q7: Do caregivers who bring their children to you - for any disease - also go to health centres during the time of that illness episode? At what stage do they come to you - before or after visiting health centres? Why?

Q8: Are there children’s illnesses that you cannot handle or manage?

(**Probes**: If yes, which ones? Why? In those cases, what advice do you give to the caregiver?)

Q9: Are you a member of any organisations of traditional healers or herbalists?

(**Probes**: Which ones? Have you attended any trainings from these organisations? If so, please tell us about those i.e. who organised them, what content was covered etc.?)

Q10: Is there anything else you would like to say, or any additional information you would like to share? Do you have any questions to ask me?

**Thank the participant for his/her time.**

**Make notes about how the discussion went; what are your impressions? Did the informant talk freely?**
